# Clinical characteristics and diagnosis of a rare case of systemic AL amyloidosis: a descriptive study

**DOI:** 10.18632/oncotarget.25055

**Published:** 2018-05-11

**Authors:** Pengguo Chen, Zhaohan Wang, Hui Liu, Donglin Liu, Zhibin Gong, Jin Qi, Jianfang Hu

**Affiliations:** ^1^ Department of Gastroenterology, Jiangxi Provincial People's Hospital, Nanchang, Jiangxi, China; ^2^ Shanghai Institute Traumatology and Orthopaedics, Ruijin Hospital, Shanghai Jiao Tong University School of Medicine, Shanghai, China

**Keywords:** amyloidosis, hepatomegaly

## Abstract

Systemic amyloidosis is a rare disease involving multiple organs. It is difficult to establish diagnosis as the symptoms is diverse and non-specific. And without specific therapy the prognosis is very poor. We analyzed detailed clinical and laboratorial data of a 53-year-old male patient. The characteristic features included refractory pleural effusion, extraordinary hepatomegaly and cardiac failure. The illness lasted 9 months and therapy period spanned 4 months. Fine needle biopsy of liver, lung, heart, pancreas and kidney was performed. Immunohistochemistry, immunofluorescence, Congo staining and hematoxylin and eosin staining were performed. All specimens were stained pink with haematoxylin and eosin staining. Amorphous deposits of eosinophilic material were visible within the Congo red dye stained liver tissue whereas under cross-polarized light pathognomonic apple-green birefringence of amyloid deposits was visible. At last systemic AL amyloidosis diagnosis was confirmed. The report showed an unusual AL amyloidosis case in detail which would be helpful for physician in clinical work.

## INTRODUCTION

The amyloidosis is rare disease that result from extracellular deposition of amyloid, a fibrillar material derived from various precursor proteins which self-assemble with highly ordered abnormal crossβ-sheet conformation [1]. The process of amyloid formation and deposition causes progressive organ dysfunction. Amyloidosis is remarkably diverse and can be hereditary or acquired, localized or systemic, and lethal or an incidental finding. 27 different human proteins with amyloidogenic potential *in vivo* have been identified; ~15 of these proteins cause systemic amyloidosis [2].

Congo staining and some sophisticated techniques such as immunoelectron microscopy and proteomic analysis are gold standard methods for identifying amyloid and its subtypes. It is difficult to diagnose amyloid in clinical work. And without effective therapy the prognosis is very poor.

## RESULTS

**Figure 1:**
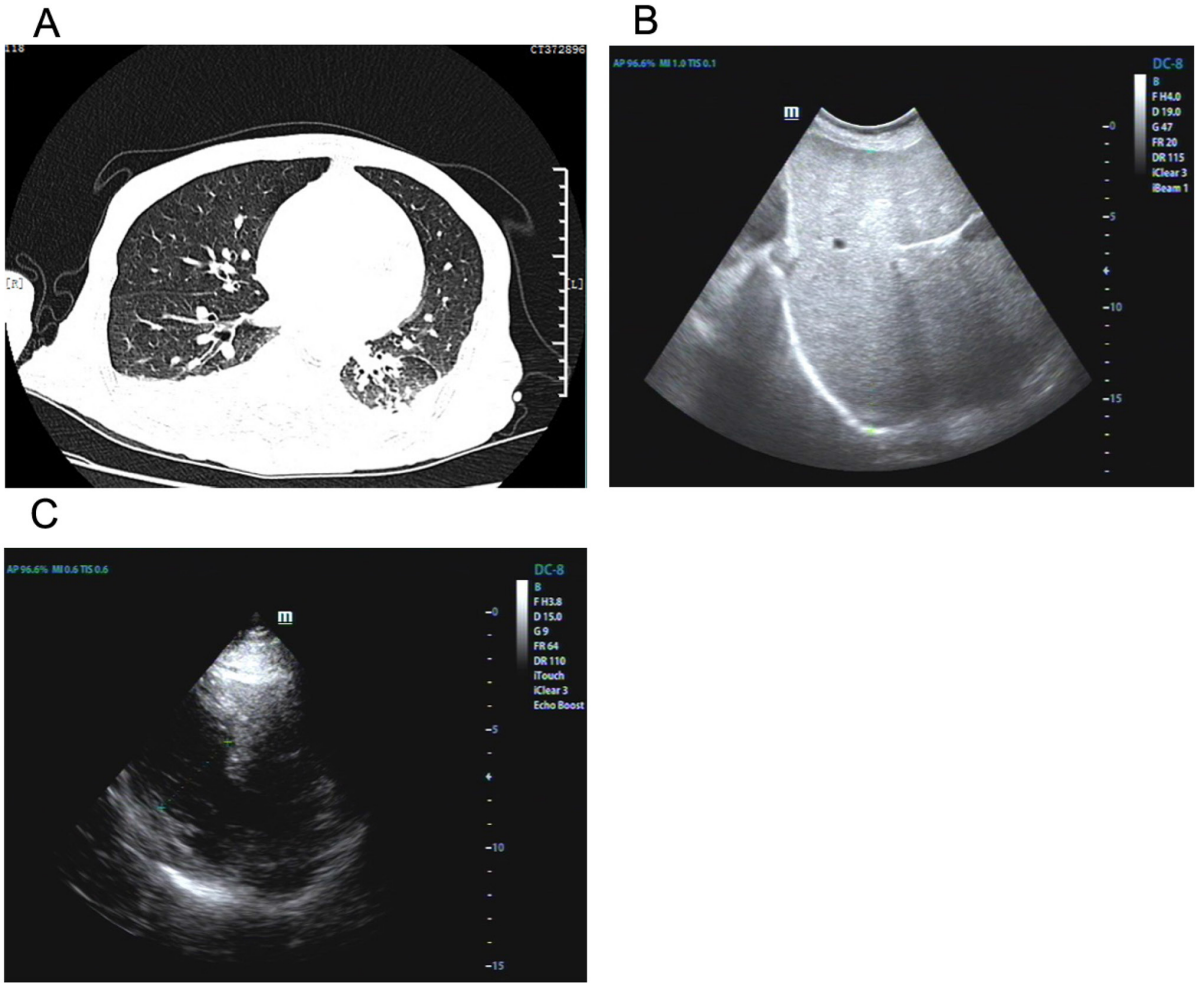
Imaging findings of lung, liver and heart Chest CT showed inflammation and pleural effusion with right basal lobe atelectasis. (**A**) Abdominal ultrasonography indicated swollen liver (**B**). Echocardiography demonstrated left ventricular wall thickness was increased (**C**).

A 53-year-old male patient was presented to a major hospital in December, 2014 due to fatigue, short of breath and weight loss lasting 6 months. Initial laboratory examinations revealed increased serum NT-proBNP level (6943 pg/ml, reference 0–300 pg/ml) and CA125 level (237.5 u/ml, reference 0–35 U/ml) whereas tuberculosis antibody was negative, routine analysis of blood and urine and ESR were within normal limits. The liver function was abnormal (Table 1). Electronic bronchoscope examination indicated upper right bronchitis. Cytological examination did not show any malignant cells. Chest CT showed pronounced bilateral pleural effusion associated with right basal lobe atelectasis and inflammation in left basilar regions (Figure 1A). Abdominal ultrasonography indicated swollen liver (Figure 1B). ECG analysis indicated T wave changes. After treated with cefotiam, levofloxacin and licorice slice for 2 weeks, the patient reported mildly clinical improvement and was discharged.

**Table 1:** Liver function in different time

	Dec 2	Jan 22	Jan 28	Feb 20	Feb 24	Mar 8	Normal range
Total protein (g/l)	55.7	62.8	60	58	64	55.8	60–88
Albumin (g/l)	40.9	44.1	37	40	43	38.2	35–64
Globulin (g/l)	14.8	18.7	23	18	21	17.6	20–35
Total bilirubin (umol/l)	26.1	40.69	49.2	80.3	99.6	144	0–17.1
Bilirubin direct (umol/l)	14.5	26.1	34.5	52.4	55.5	120	0–6.8
Glutamic-pyruvic transaminase (U/L)	8	17	17	27	29	258	0–45
Glutamic-oxaloacetic transaminase (U/L)	18	31	28	41	38	861	0–40
Glutamyltranspeptidase (U/L)	295	191	182	75	163	365	0–50
Alkaline phosphatase (U/L)	163	153	116	153	125	230	40–150

The patient was readmitted to another major hospital a month later on Jan 21. Initial laboratory evaluation revealed the presence of tuberculosis serum antibody, hepatitis B, hepatitis C, HIV infection and treponema pallidum antibody were negative and CEA, AFP, CA199 and IFN-γwere all within normal ranges. Serum CA125 level got higher (>1000 U/ml, reference 0–35 U/ml) and liver functions got worsen (Table 1). Chest CT examination revealed patchy shadow and a small amount of pleural effusion on the right side. Abdominal contrast-enhanced MRI showed swollen liver, portal edema and pleural effusion. There was no significant improvement after treated with ceftazidime, ursodeoxycholic acid, isosorbide mononitrate for 10 days

The patient was referred to the same hospital in February 19. He was anorexia, emaciated, hypodynamia and tachypnea. The presence of C-ANCA, p-ANCA, PR3-ANCA, MPO-ANCA, Rn/A, Ro-52, SS-B, Scl-70, PM-Scl, Jo-1, CENP-B, PCNA, ds-DNA, ANuA, Histone, Rib-P, AMA-M2 antibodies were negative. The symptom of xanthochromia aggravated. The patient was diagnosed as liver cancer or lymphoma and was treated with ursodeoxycholic acid, ginseng, aspartate ornithine and mannitol.

On February 23, the patient was transferred to our hospital. Examination of the abdomen revealed an enlarged liver extending 20 cm below the right costal margin at the midclavicular line. ECG examination indicated atrial fibrillation later. Echocardiography demonstrated both ventricles were normal in size along with left ventricular wall thickness increased, ejection fraction reduced (43%) and left atrium enlarged (Figure 1C). ELISA tests for toxoplasma, cysticercus, paragonimus westermani and schistosome were negative. Serum test for copper blue protein, serum ferritin, CMV and autoimmune hepatitis were within limits. Liver function deteriorated (Table 1). After treated with Magnesium isobutyrate, adenosine methionine, tanshinone, the condition declined sharply and the patient died of cardiac arrest 20 days later.

**Figure 2:**
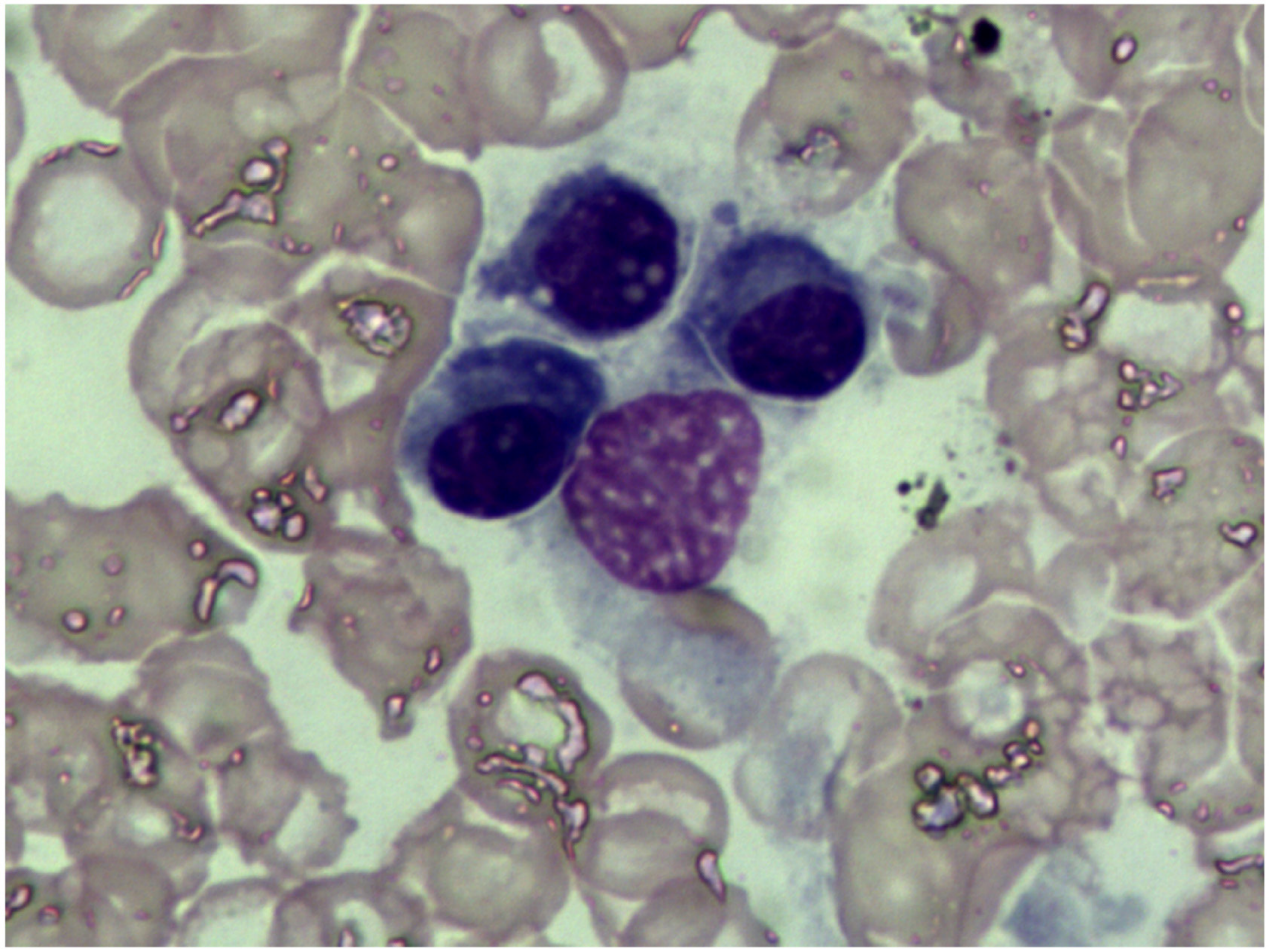
Bone marrow cytology examination The percentage of plasma cell was within ranges (3.25%) and eosinophil granulocytes were easily noted.

**Figure 3:**
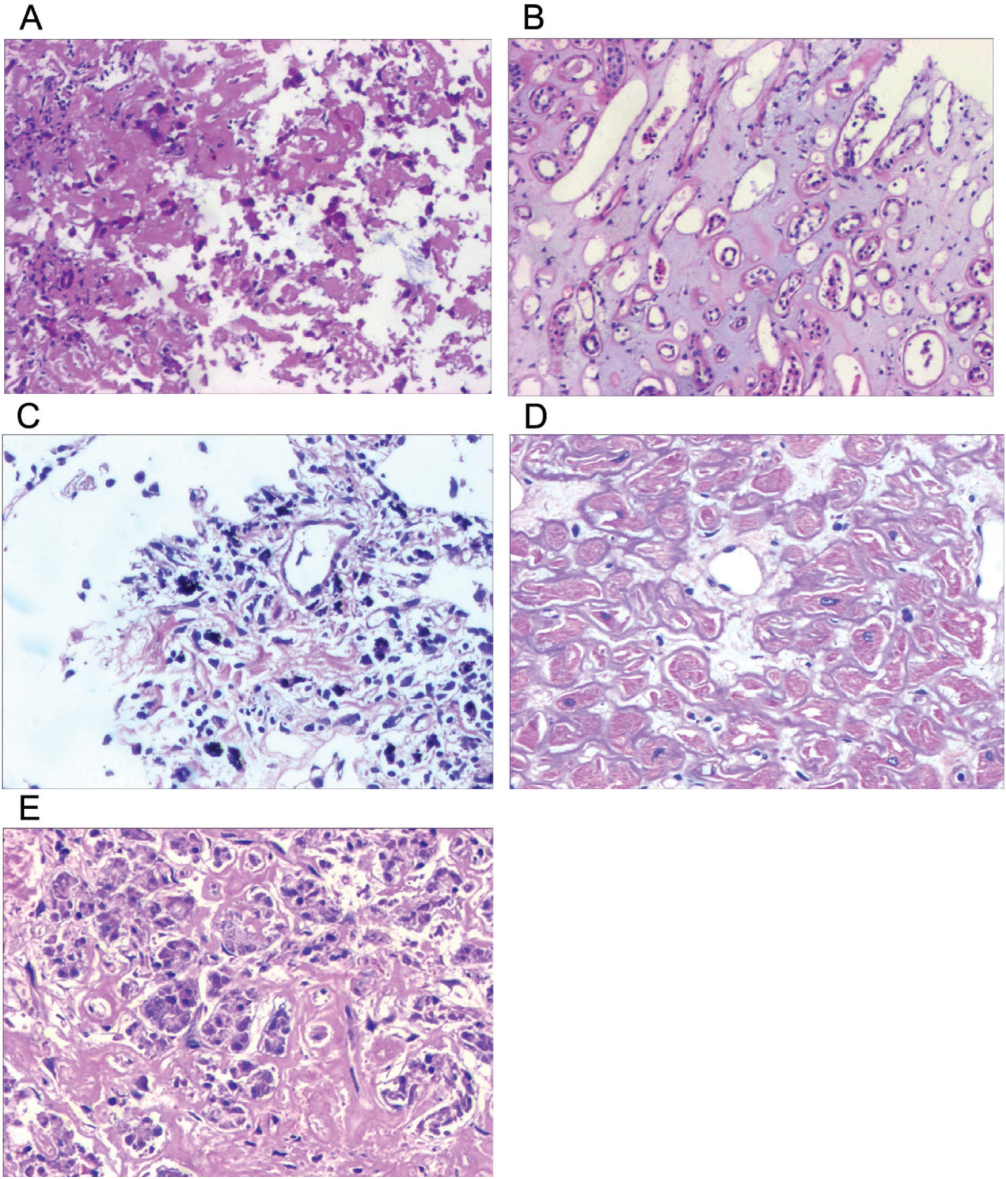
Hematoxylin and eosin staining of multiple organs There is amorphous extracellular material persent in sorts of tissue from liver (**A**), kidney (**B**), lung (**C**), heart (**D**) and pancreas (**E**). (magnification ×20).

**Figure 4:**
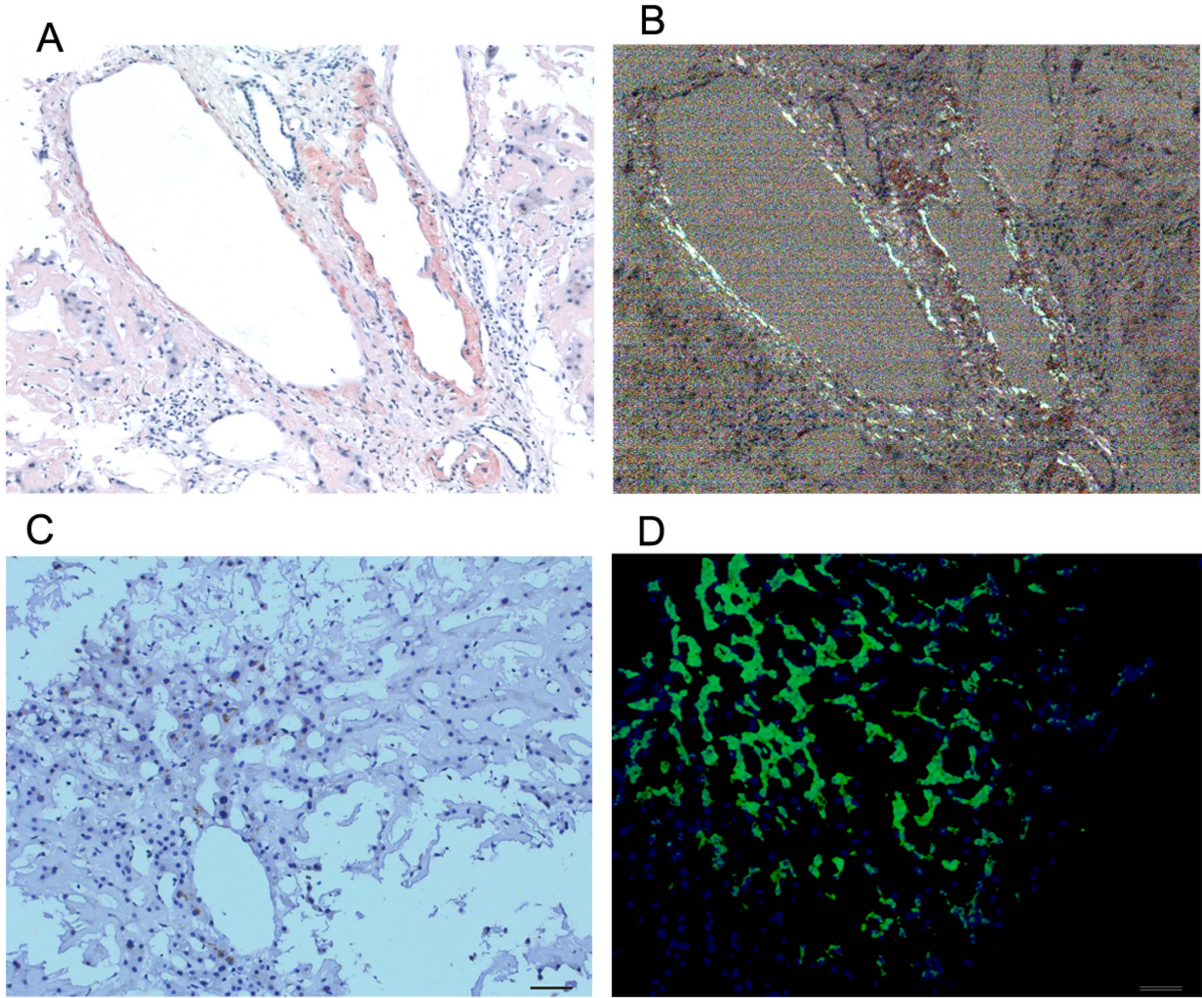
Histological findings on liver tissue Congo red staining of tissue biopsy, the gold standard for confirming amyloid deposition, a pink-orange color under lightmicroscope (**A**) and typical apple green briefringence under cross-polarised light (**B**). Imminohistochemical staining (**C**) by antibodies to serum amyloid P (SAP). Immunofluorescence analysis (**D**) showed bright green fluorescence using the novel luminescent conjugated polymer pentameric formicthiophene acetic acid (pFTAA). (magnification ×20).

Bone marrow findings revealed the percentage of plasmacell was within ranges (3.25%) and eosinophil granulocytes were easily noted (Figure 2). Fine needle biopsy of liver, lung, heart, pancreas and kidney was performed. All specimens were stained pink with haematoxylin and eosin (Figure 3). Amorphous deposits of eosinophilic material was visible within the Congo red dye stained liver tissue which has a pink-orange color. And pathognomonic apple-green birefringence of amyloid deposits was visible when viewed under cross-polarized light (Figure 4A, 4B). Serum amyloid P (SAP) is component of all amyloidosis. Immunohistochemistry for SAP antibody confirmed staining in the liver tissue (Figure 4C). Immunofluorescence analysis (Figure 4D) showed bright green fluorescence exhibited by hepatic amyloid deposits using the novel luminescent conjugated polymer pentameric formicthiophene acetic acid (pFTAA).

## DISCUSSION

The amyloidosis is rare disease that result from extracellular deposition of amyloid which contain several minor non-fibrillary constituents, including glycosaminoglycans (GAGs) and serum amyloid P component (SAP). The specific, highly ordered ultrastructure of amyloid fibrils accounts for their characteristic property of binding Congo red dye in a spatial manner that produces green birefringence when viewed under cross-polarized light. This birefringence remains the histological gold standard for confirming the presence of amyloid in tissue [1]. The most frequent syndromes are heart failure, nephrotic syndrome, hepatomegaly and peripheral neuropathy [3]. A stepwise approach to diagnosis and staging of amyloidosis is critical and includes confirmation of amyloid deposition and identification of fibril type [4].

Amyloidosis should be suspected in any patient with the following symptoms: nondiabetic albuminuria, non-ischemic cardiomyopathy in absence of primary heart or renal disease. Before admitted to hospitals, the patient presented anorexia, emaciated, hypodynamia and tachypnea for 5 months. Cardiomyopathy, hepatomegaly and liver dysfunction was observed. A pink-orange color was noted within the Congo red dye stained liver tissue. And pathognomonic apple-green birefringence was viewed under cross-polarized light. Further, immunohistochemistry for SAP showed staining in the liver tissue. Those manifestations were accordance with amyloidosis.

Amyloidosis was classified to several subtypes, such as AL amyloidosis, AA amyloidosis, hereditary amyloidosis, etc. AL amyloidosis is involved in the kidney, heart, liver, pleura, lungs, colon and the autonomic nervous system [5]. Our immunofluorescence analysis showed bright green fluorescence exhibited by hepatic amyloid deposits using the novel luminescent conjugated polymer pentameric formicthiophene acetic acid (pFTAA) which may be helpful for AL amyloidosis diagnosis [6]. AA amyloidosis is mainly induced by tuberculosis, chronic inflammation and tumor. Proteinuria or renal dysfunction is a characteristic feature of AA amyloidosis. Hepatosplenomegaly owing to amyloid infiltration is seen at presentation in 9% of AA amyloidosis patients whereas liver failure is exceptionally rare [7]. The hereditary systemic amyloidosis usually presents with renal dysfunction in their 6th or 7th decade [3, 8]. Subsequently our constellation finding was compatible with AL amyloidosis diagnosis.

Typical clinical manifestations of AL amyloidosis include proteinuria and nephrotic syndrome, cardiomyopathy, hepatomegaly, neuropathy [9]. Cardiac involvement is the leading cause of mortality in amyloidosis. Amyloid deposition in the heart typically presents as restrictive cardiomyopathy while low cardiac output [10–11]. In this case echocardiography showed left ventricular wall thickness increased associated with mitral valve thickened. When an echocardiogram shows thickening of the ventricular myocardium without hypertension amyloidosis should be considered [5, 12]. Atrial and ventricular arrhythmias are manifestations of cardiac amyloidosis [2]. Our Electrocardiogram records indicated S-T change and atrial fibrillation later.

Pleural effusions may result from amyloid heart disease or pleural amyloid deposition [13]. In the patient, the refractory nature of the effusions with a poor response to diuretics – is suggestive of pleural involvement. The interstitial opacities revealed on chest CT reflected parenchymal lung involvement.

Hepatic amyloid is present in ~50% of amyloidosis patients. The examination of the abdomen and abdominal CT demonstrated an extraordinarily enlarged liver. Liver function is often well preserved with modest increase in ALP (alkaline phosphatase) and GGT (gamma-glutamyl transpeptidase) levels [2]. The marked elevation of ALP and GGT is characteristic of hepatic amyloidosis [14]. Hepatomegaly and liver function results were consistent with hepatic amyloidosis in the case.

The amyloidosis is classified according to the amyloidogenic protein. In AL amyloidosis, the protein is an immunoglobulin light chain which is produced by a clone of plasma cell. The plasma-cell burden is usually low [15–16]. The bone marrow analysis in the case indicated the amount of plasma cells was very modest (3.5%, median 10%).

Various variables are powerful clinical indicators of poor outcome, including poor performance status, severe postural hypo-tension [17]. Patients with NT-proBNP>8500 ng/l had median survival of 3 months [18]. Patients with higher concentrations of FLC at diagnosis also have poorer outcomes with bone marrow plasmacytosis greater than 10% [19–20]. NT-proBNP level was more than 7000 ng/l and the symptoms of hypodynamia and tachypnea deteriorated which suggested a poor outcome in the case.

Hyperbilirubinaemia is unusual but is associated with poor outcomes as well [2]. In this case the levels of TBIL (total bilirubin) and DBIL (direct bilirubin), ALP and GGT increased gradually. TBIL and DBIL climbed to 144 and 122 umol/l respectively.

In clinical work amyloidosis is not easy to diagnose. Immunoelectron microscopy with gold-labelled anti-fibril protein antibodies is very sensitive. Proteomic analysis using mass spectrometry is a discriminating and sophisticated technique and new gold standard for fibril typing. SAP scintigraphy, a specific imaging method, enables the amyloid load to be ascertained and monitored serially [1]. Treatment of AL amyloidosis usually comprises chemotherapy and autologous stem cell transplant (ASCT) [21]. SAP is another attractive therapeutic target as it binds to all types of amyloid fibrils and protects them from proteolytic cleavage. Depletion of SAP might enhance amyloid clearance or slow amyloid formation *in vivo* [1].

The patient had a long illness history. Clinical records indicated the presence of refractory pleural fluid, cardiomyopathy and hepatomegaly. Distinctive from common amyloidosis, some characteristic features such as proteinuria, nephrotic syndrome, macroglossia and periorbital purpura were absent. Histological staining analysis demonstrated that liver, lung, heart, pancreas and kidney probably involved in amyloid course. To our knowledge, there is no such report as yet.

As the symptoms were varied. For several months tuberculous pleurisy, viral hepatitis, liver cancer or lymphoma was suspected. And the treatment did not work effectively while several diseases rather than a specific disease were thought at one time. Especially when sorts of therapy failed a single etiology should be considered. Often multidisciplinary consultation was necessary. Due to the limited availability of sophisticated technique it is still a huge challenge to identify amyloidosis particularly in early stage. In this case routine examination is not enough. Some available examination such as mass spectrometry or PET-CT would be valuable. The diagnosis was built based on histopathologic examinations. But it was so late to perform tissue biopsy that specific treatment chance was lost.

On our study there are still some limitations for instance the lack of mass spectrometry analysis owning to scarcity of specimen. There is also confusion on the highest level of CA125 in serum. CA125 is a repeating peptide episode of the mucin MUC16, which promotes cancer cell proliferation and inhibits anti-cancer immune responses. CA125 is widely used as a diagnostic marker for ovarian cancer [22]. In this male patient, tumors were ruled out. And we can't interpret the results.

## MATERIALS AND METHODS

### Patient and tissue analysis

We reported an unusual amyloidosis case with multiple organs involvement. The medical records in several major hospitals spanning across four months were compiled and reviewed by our team. Liver biopsy was performed. And autopsy specimens of heart, lung, kidney and pancreas were obtained after the patient's death. All experimental protocols were approved by the Ethics Committee of Jiangxi Provincial People's Hospital. Written informed consent was obtained from the close contacts. Hematoxylin-eosin (HE) staining, Congo red staining for tissue and immunohistochemistry and immunofluorescence was performed as described previously [23–25].
